# Public Willingness to Undergo Presymptomatic Genetic Testing for Alzheimer's Disease

**DOI:** 10.1155/2019/2570513

**Published:** 2019-03-03

**Authors:** Mohammed H. Alanazy, Khalid A. Alghsoon, Abdulaziz F. Alkhodairi, Faisal K. Binkhonain, Turkey N. Alsehli, Feras F. Altukhaim, Ibrahim M. Alkhodair, Taim Muayqil

**Affiliations:** Division of Neurology, Department of Internal Medicine, King Saud University Medical City and College of Medicine, King Saud University, Riyadh, Saudi Arabia

## Abstract

Presymptomatic genetic testing (PGT) for Alzheimer's disease (AD) is available for at-risk individuals. This study aimed to investigate the public perception of PGT in Saudi Arabia and determine variables that might influence the decision to undergo PGT. A questionnaire link was posted on Twitter by the Saudi Alzheimer's Disease Association and was made publicly available on social media networks. A total of 2935 people participated, of which 59.9% were willing to undergo PGT. Of these, 26.8% reported having a family history of AD, and 0.24% had two family members with early onset AD. The reasons cited for willingness to undergo PGT included the following: to adopt a healthier lifestyle, to ensure appropriate family and financial planning, to seek early treatment, and to relieve anxiety. In multiple logistic regression analysis, willingness to undergo PGT was negatively associated with having a self-reported family history of dementia (OR 0.81, 95% CI 0.68-0.96) and was positively associated with marital status (OR 1.39, 95% CI 1.13-1.70). In conclusion, PGT for AD seems to be well accepted in this large Saudi cohort. The reasons cited are similar to those reported elsewhere in the literature.

## 1. Introduction

Alzheimer's disease (AD), the most common form of dementia, presents as a progressive decline in cognition, behavior, and functional abilities and develops as a consequence of aggregation of *β*-amyloid and hyperphosphorylated tau in vulnerable brain regions [1]. The cognitive domains affected by AD mostly involve short-term memory, visuospatial function, executive function, and language. The diagnosis of AD is typically made on the basis of clinical and cognitive assessment; however, recent advances in neuroimaging, cerebrospinal fluid biomarkers, and genetic testing can facilitate early and presymptomatic diagnosis. The predominant form of the disease is late-onset AD (LOAD), which usually manifests at or after 65 years of age. Early onset AD (EOAD) accounts for less than 5% of cases and has an age of onset below 65 years. A fully penetrant autosomal dominant form of EOAD is caused by mutations in the amyloid precursor protein (*APP*) and presenilin-1 (*PSEN1*) genes, whereas the penetrance of mutations in presenilin-2 (*PSEN2*) gene is 95% [2]. In contrast, individuals with one copy of the apolipoprotein E (*APOE*)∗*E4* allele have a three- to fourfold increase in the risk of developing AD, particularly at an older age [2, 3]. The risk is higher in individuals who have two copies of the* APOE∗E4* allele [2, 3]. However,* APOE* is not a deterministic gene but rather considered a susceptibility gene, and the risk conferred by the* E4* allele differs based on age, sex, and ethnicity [2, 3]. Due to its limited clinical utility, genetic testing for* APOE* is not recommended [2].

Identification of dominantly inherited genes and the revolution in genomic technology has made genetic testing readily available for diagnostic purposes in symptomatic individuals and for predictive purposes in asymptomatic individuals with a family history of AD [2]. Identifying AD mutation carriers in the presymptomatic stage facilitates enrollment in preclinical treatment trials, with the ultimate goal of initiating therapy before the manifestation of irreversible brain damage [4]. Presymptomatic genetic testing (PGT) should always be preceded by genetic counseling and is preferably performed on the basis of a positive result in an affected family member [2]. Caselli et al. reported that an online web-based survey revealed that 80.8% of respondents were willing to undergo PGT for AD [5]. The population of that study was limited to participants who were interested in AD prevention research and had registered with an AD prevention registry website, which likely explains the high willingness to undergo PGT. In addition, public perception of PGT may vary across locations and may be influenced by cultural and religious beliefs of a society. Thus, this study sought to assess public perception of PGT for AD in Saudi Arabia and explore factors that might influence willingness to participate in genetic testing. 

## 2. Methods

This study was approved by the institutional review board of King Saud University. A questionnaire link was posted on Twitter by the Saudi Alzheimer's Disease Association (approximately 38,000 followers) and was made publicly available on social media networks during the period from April to August 2018. Participants were encouraged to forward the survey link to their social networks, resulting in a snowball sample. To prevent duplicate responses, the survey was programmed to allow only one response per IP address (using surveymonkey.com).

The first draft of the questionnaire was developed by all authors in Arabic, based on literature review and taking into consideration the general culture of the Saudi society. The questionnaire was subsequently reviewed by 2 neurologists for completeness and comprehension. A group of randomly selected healthy volunteers of different ages and educational levels were asked to assess the coherency and readability of the questionnaire. In total, 3 successive versions of the questionnaire were tested in 20 healthy volunteers before it was finalized. The questionnaire was structured into the following sections: (1) an introductory statement briefly explaining the disease with emphasis on the current absence of disease-modifying therapy, (2) informed consent where participants consented if they select “agree to participate” button, (3) participant demographics, (4) questions regarding family history of AD or dementia, (5) a question related to the participant's experience of being a caregiver for a patient with AD, (6) questions regarding the participant's willingness to undergo PGT for AD, and (7) the participant's reasons for undergoing or rejecting PGT (maximum of 3). The questions regarding PGT were structured to be self-explanatory, e.g., for autosomal dominant AD genes, “if the gene being tested is inherited in a dominant manner and its presence means you will certainly develop AD at a young age (generally before 50 years), would you agree to do this test?” and for* APOE* “if the gene being tested increases your risk of developing AD at an older age (generally after 65 years) but without certainty (as there are people who have this gene and do not develop AD), would you agree to do this test?”

## 3. Analysis

Demographic and categorical data were reported as numbers and percentages. Multiple logistic regression analysis was employed to determine characteristics associated with a decision to undergo or reject PGT for AD. Independent variables included age, sex, marital status, highest level of education, having a first- or second-degree relative with dementia, and having been a caregiver for a patient with dementia. An odds ratio (OR) and 95% confidence interval (CI) were calculated for each independent variable. A two-sided P value < 0.05 was considered significant. Data analysis was conducted using statistical software SPSS, version 23 (IBM, Armonk, NY).

## 4. Results

Of 3060 participants, 2935 (55% women, 45% men) returned complete questionnaires. Incomplete questionnaires were not included in the analysis.  Table 1 shows the demographics and characteristics of the participants. The majority of participants were 18-30 years old (39.5%), married (59.9%), living in Riyadh (66.4%), of Saudi nationality (94.7%), and had a bachelor's degree as their highest level of education (52.7%).

Table 2 shows participants' family histories of AD and dementia. Family history of a first- or second-degree relative with AD was reported by 786 participants (26.8%). Family history of having both a first- and second-degree relative with AD was reported by 161 participants (5.5%), 7 (0.24%) of whom reported that the onset was before 50 years of age. Of all participants, 526 (17.9%) reported having been a caregiver for a patient with AD.

Regarding participants' willingness to have PGT for AD, 59.9% were willing to undergo PGT for either the autosomal dominant genes or the APOE, whereas 40.1% rejected both tests (Table 3). The most frequently selected reasons for accepting PGT (1758 participants; Table 4) were “to adopt a healthy lifestyle” (52.1%), “to make plans for future and family planning” (41.5%), “possibility of finding a cure for the disease in the future” (40.0%), and “to relieve anxiety” (37.8%). A minority (4.7%) provided descriptive reasons that were mostly related to “having forgetfulness” and “a desire to participate in research.” Of those who expressed willingness to undergo PGT, 76.5% indicated that their acceptance would be contingent upon the test being offered free. The most frequently selected reasons for rejecting PGT (1177 participants, Table 5) were “worried about developing a mental illness after knowing the results” (32.5%), “fear of the result of genetic testing” (27.7%), and “absence of cure” (22.9%). A minority (5.8%) provided descriptive reasons that were mostly related to spiritual faith and an absence of family history of dementia.

In the logistic regression analysis, only two factors were found to contribute significantly to participants' willingness to undergo PGT. Participants with family history of a first- or second-degree relative with dementia were less likely to accept PGT (OR=0.81, 95% CI=0.68-0.96, and P=0.02), and married participants were more likely to accept PGT (OR=1.39, 95% CI=1.13-1.70, and P=0.002).

## 5. Discussion

This is the first study on public perception of PGT for AD in Saudi Arabia and one of the few studies undertaken to date worldwide. In this study, more than half of participants showed willingness to undergo PGT for AD. Married individuals are more likely to accept PGT testing, while those with a family history are less likely to do so. A conjectural explanation is that married individuals may want to know their risk of developing AD to help them with family planning (e.g., having children). In contrast, individuals who have a relative with dementia may not want to know the risk to avoid the emotional and psychological consequences of knowing that their health will deteriorate similarly to that of their loved ones. In contrast, Caselli et al. reported that male sex and number of affected relatives are associated with a higher likelihood of agreeing to PGT and higher education is associated with a lower likelihood [5]. This discrepancy between the two studies may be influenced by the differences in religion, culture, beliefs, and values of the studied populations. In Islam, premarital genetic testing is considered an acceptable procedure [6]. The high frequency of consanguineous marriage in Saudi Arabia is one of the cultural factors that contributes to the high prevalence of inherited hemoglobinopathies [7] and, conceivably, to the increased risk of other autosomal recessive diseases (AR). Therefore, the premarital screening and genetic counselling program (PMSGC) has been established and made mandatory to reduce the burden of AR diseases, specifically sickle-cell disease and *β*-thalassemia [7]. The PMSGC has been successful in reducing the prevalence of *β*-thalassemia [7]. Yet, 48% of at-risk marriages were completed in 2009, indicating that the cultural challenges were not fully addressed by the genetic counselors [7].

Secondly, the discrepancy between our study and the study by Caselli et al. may be related to differences in the participant samples. In our study, the survey link was available to all adults in the community, whereas in that study by Caselli et al., it was confined to those registered with an AD prevention registry website [5]. Other studies of at-risk individuals reported that 54% accepted PGT for neurodegenerative diseases [8], and 58% accepted PGT for Huntington's disease and autosomal dominant spinocerebellar ataxia [9]. Notably, the number of eligible individuals seeking PGT typically drops following genetic counseling, reflecting the complexity of the decision-making process [10, 11].

Common reasons cited for unwillingness to undergo PGT in this and previous studies include fear of the consequences of the results [5], absence of curative therapy [8], and fear of the psychological impact [5]. Despite the current absence of disease modifying or preventative therapy, most at-risk individuals that are tested for hereditary ataxias and neuromuscular disorders find PGT beneficial and cope well regardless of the results. However, the impact of PGT results on anxiety and depression is unpredictable [12]. Generally, even individuals with normal test results experience conflicting emotions of happiness, guilt, relief, fear, and anger [13]. While psychological support is required for all PGT seekers [14], some may experience severe distress after receiving a result [15] and require intensive support from health professionals experienced in counseling.

Slooter et al. [16] reported that 25% of the general population aged 55 years and older have a first-degree relative with dementia. Our results demonstrate a lower rate (18.3%), perhaps because the majority of our participants were younger (Tables 1 and 2). However, in this study, a notable proportion of the participants reported having a first- or second-degree relative with AD (26.8%) and dementia (38.6%). While this study was not designed to assess the prevalence of dementia in Saudi Arabia, and reported diagnoses were not investigated, our data may reflect a high prevalence rate of AD and dementia that is not unexpected in view of the population growth and aging, the high illiteracy rate among people older than 65 years [17], and the high prevalence of cardiovascular risk factors [18]. Using a validated Arabic version of the Montreal cognitive assessment test (MOCA), a previous study reported a prevalence of dementia of 26% among Saudis attending primary care clinics, which decreased to 6.4% after using an education-adjusted MOCA score [19]. However, those data were obtained by convenience sampling; more representative samples would be required to better estimate the population prevalence of AD and dementia in Saudi Arabia.

Autosomal dominant AD has a younger age of onset compared with sporadic LOAD, but variations have been observed within families and various mutation types [20]. We decided to use the age of 50 years (rather than 60 years) to define young onset AD. This was supported by a meta-analysis of symptom onset in autosomal dominant AD that estimated a mean age of onset to be 46.2 years [20]. In addition, we took into consideration the Saudi culture, whereby people cite their age using the Hijri calendar in which a person's age is older by approximately one year for every 30 years in the Gregorian calendar. Most older people may not know their exact date of birth either because they were born in a rural-Bedouin society or born prior to the development of stringent governmental birth record keeping. For these reasons, older Saudi Arabians may not report their age, or the age of their elderly relatives, accurately. Therefore, we decided to choose a more conservative age limit when identifying EOAD.

EOAD has been estimated to comprise 1-5% of all AD cases [21], and autosomal dominant AD accounts for 1-2% [4]. Among all participants in this study, 5.5% reported having both a first- and second-degree relative with AD, and 0.24% reported that the onset was before the age of 50 years. Notably, these data are based on information about diagnosis and age of onset as perceived by the participants, rather than as ascertained by a physician. Nonetheless, it is reasonable to consider those participants who reported having 2 family members with EOAD to have a potential risk for AD.

In addition to the discussed limitations above, although the questionnaire link was open to all adults from the society, sampling bias cannot be excluded. It is possible that there was a bias in participation from people who are interested in or have a family history of AD. Furthermore, input from people who do not have access to internet and social media, especially those older than 50 years, was limited due to our data collection method.

In conclusion, willingness to undergo PGT was expressed by approximately 60% of our study population. Significant associations were observed between participants' willingness to undergo PGT and their marital status as well as self-reported family history of dementia. In addition, the study explored variables that might influence the decision to undergo PGT. Pending further prospective studies, these findings may be helpful to physicians to develop a general framework when counseling relatives of patients with AD.

## Figures and Tables

**Table 1:** Characteristics of the study population (total number of participants =2935).

Participants	Number (%)

Sex	
Female	1614 (55.0)
Male	1321 (45.0)

Age	
18-30 years	1158 (39.5)
31-40 years	749 (25.5)
41-50 years	597 (20.3)
51-60 years	336 (11.4)
> 60 years	95 (3.2)

Marital status	
Married	1757 (59.9)
Not married (single, divorced, widowed)	1178 (40.1)
Nationality	
Saudi	2780 (94.7)
Non-Saudi	155 (5.3)

City of residence	
In Riyadh	1949 (66.4)
Out of Riyadh	986 (33.6)

Highest level of education	
No schooling	13 (0.4)
Primary school	32 (1.1)
Secondary school	67 (2.3)
High school	615 (21.0)
Diploma	286 (9.7)
Bachelor's degree	1546 (52.7)
Master's degree	259 (8.8)
PhD	117 (4.0)

**Table 2:** Participants' report of family history of Alzheimer's disease, dementia, and age of onset before 50 years (total number of participants =2935).

Participants' report	Number (%)	Onset before 50 years of age; number (%)

First-degree relative with AD	363 (12.4)	24 (0.82)
Second-degree relative with AD	584 (19.9)	19 (0.53)
Both first- and second-degree relatives with AD	161 (5.5)	7 (0.24)
First- or second-degree relative with AD	786 (26.8)	NA
First-degree relative with dementia	536 (18.3)	33 (1.12)
Second-degree relative with dementia	870 (29.6)	28 (0.95)
Both first- and second-degree relatives with dementia	273 (9.3)	11 (0.37)
First- or second-degree relatives with dementia	1133 (38.6)	NA
Being a caregiver for a patient with AD	526 (17.9)	NA

**Table 3:** Participants' perception about presymptomatic genetic testing for Alzheimer's disease (total number of participants =2935).

Participants' perception about presymptomatic genetic testing for autosomal dominant AD genes, APOE, or both.	Number (%)

Accepting either genetic tests	1758 (59.9)
Accepting both genetic tests	1325 (45.1)
Accepting autosomal dominant AD genetic test	1560 (53.2)
Accepting APOE gene test	1523 (51.9)
Rejecting both tests	1177 (40.1)

**Table 4:** Reasons for accepting presymptomatic genetic testing for Alzheimer's disease (total responses 1758).

Reasons	Number of responses (%)

To adopt a healthy lifestyle	916 (52.1%)
To make plans for future and family planning	730 (41.5%)
The possibility of finding a cure for Alzheimer's disease in the future	703 (40.0%)
To relieve anxiety	664 (37.8%)
To make plans regarding my job and financial planning	396 (22.5%)
I know a person with Alzheimer disease	221 (12.6%)
Other	82 (4.7%)

**Table 5:** Reasons for rejecting presymptomatic genetic testing for Alzheimer's disease (total responses 1177).

Reasons	Number of responses (%)

Worried about developing a mental illness after knowing the result	383 (32.5%)
Fear of the result of genetic testing	326 (27.7%)
No cure for Alzheimer disease currently	269 (22.9%)
Worried that the result will affect my employment	116 (9.9%)
I do not trust genetic tests	104 (8.8%)
Afraid from financial consequences of the medical care	33 (2.8%)
Worried about discrimination by insurance companies	31 (2.6%)
Other	68 (5.8%)

## Data Availability

The data used to support the findings of this study are available from the corresponding author upon request.
